# Challenges of teachers when teaching sentence-based mathematics problem-solving skills

**DOI:** 10.3389/fpsyg.2022.1074202

**Published:** 2023-02-01

**Authors:** Albert Nguong Baul Ling, Muhammad Sofwan Mahmud

**Affiliations:** Faculty of Education, Universiti Kebangsaan Malaysia, Bangi, Malaysia

**Keywords:** mathematics, problem solving, teaching challenges, teaching approaches, primary school

## Abstract

Sentence-based mathematics problem-solving skills are essential as the skills can improve the ability to deal with various mathematical problems in daily life, increase the imagination, develop creativity, and develop an individual’s comprehension skills. However, mastery of these skills among students is still unsatisfactory because students often find it difficult to understand mathematical problems in verse, are weak at planning the correct solution strategy, and often make mistakes in their calculations. This study was conducted to identify the challenges that mathematics teachers face when teaching sentence-based mathematics problem-solving skills and the approaches used to address these challenges. This study was conducted qualitatively in the form of a case study. The data were collected through observations and interviews with two respondents who teach mathematics to year four students in a Chinese national primary school in Kuala Lumpur. This study shows that the teachers have faced three challenges, specifically low mastery skills among the students, insufficient teaching time, and a lack of ICT infrastructure. The teachers addressed these challenges with creativity and enthusiasm to diversify the teaching approaches to face the challenges and develop interest and skills as part of solving sentence-based mathematics problems among year four students. These findings allow mathematics teachers to understand the challenges faced while teaching sentence-based mathematics problem solving in depth as part of delivering quality education for every student. Nevertheless, further studies involving many respondents are needed to understand the problems and challenges of different situations and approaches that can be used when teaching sentence-based mathematics problem-solving skills.

## 1. Introduction

To keep track of the development of the current world, education has changed over time to create a more robust and effective system for producing a competent and competitive generation (Hashim and Wan, 2020). The education system of a country is a significant determinant of the growth and development of the said country (Ministry of Education Malaysia, 2013). In the Malaysian context, the education system has undergone repeated changes alongside the latest curriculum, namely the revised Primary School Standard Curriculum (KSSR) and the revised Secondary School Standard Curriculum (KSSM). These changes have been implemented to ensure that Malaysian education is improving continually so then the students can guide the country to compete globally (Adam and Halim, 2019). However, Malaysian students have shown limited skills in international assessments such as Trends in International Mathematics and Science Study (TIMSS) and the Program for International Student Assessment (PISA).

According to the PISA 2018 results, the students’ performance in mathematics is still below the average level of the Organization for Economic Co-operation and Development (OECD; Avvisati et al., 2019). The results show that almost half of the students in Malaysia have still not mastered mathematical skills fully. Meanwhile, the TIMSS results in 2019 have shown there to be a descent in the achievements of Malaysian students compared to the results in 2015 (Ministry of Education Malaysia, 2020b). This situation is worrying as most students from other countries such as China, Singapore, Korea, Japan, and others have a higher level of mathematical skills than Malaysian students. According to Mullis et al. (2016), these two international assessments have in common that both assessments test the level of the students’ skills when solving real-world problems. In short, PISA and TIMSS have proven that Malaysian students are still weak when it comes to solving sentence-based mathematics problems.

According to Hassan et al. (2019), teachers must emphasize the mastery of sentence-based mathematics problem-solving skills and apply it in mathematics teaching in primary school. Sentence-based mathematics problem-solving skills can improve the students’ skills when dealing with various mathematical problems in daily life (Gurat, 2018), increase the students’ imagination (Wibowo et al., 2017), develop the students’ creativity (Suastika, 2017), and develop the students’ comprehension skills (Mulyati et al., 2017). The importance of sentence-based mathematic problem-solving skills is also supported by Ismail et al. (2021). They stated that mathematics problem-solving skills are similar to high-level thinking skills when it comes to guiding students with how to deal with problems creatively and critically. Moreover, problem-solving skills are also an activity that requires an individual to select an appropriate strategy to be performed by the individual to ensure that movement occurs between the current state to the expected state (Sudarmo and Mariyati, 2017). There are various strategies that can be used by teachers to guide students when developing their problem-solving skills such as problem-solving strategies based on Polya’s Problem-Solving Model (1957). Various research studies have used problem-solving models to solve specific problems to improve the students’ mathematical skills. Polya (1957), Lester (1980), Gick (1986), and DeMuth (2007) are examples. One of the oldest problem-solving models is the George Polya model (1957). The model is divided into four major stages: (i) understanding the problem; (ii) devising a plan that will lead to the solution; (iii) Carrying out the plan; and (iv) looking back. In contrast to traditional mathematics classroom environments, Polya’s Problem-Solving Process allows the students to practice adapting and changing strategies to match new scenarios. As a result, the teachers must assist the students to help them recognize whether the strategy is appropriate, including where and how to apply the technique.

In addition, problem-solving skills are one of the 21st-century skills that need to be mastered by students through education now so then they are prepared to face the challenges of daily life (Khoiriyah and Husamah., 2018). This statement is also supported by Widodo et al. (2018) who put forward four main reasons why students need to master problem-solving skills through mathematics learning. One reason is that sentence-based mathematics problem-solving skills are closely related to daily life (Wong, 2015). Such skills can be used to formulate concepts and develop mathematical ideas, a skill that needs to be conveyed according to the school’s content standards. The younger generation is expected to develop critical, logical, systematic, accurate, and efficient thinking when solving a problem. Accordingly, problem solving has become an element that current employers emphasize when looking to acquire new energy sources (Zainuddin et al., 2018). This clearly shows that problem-solving skills are essential skills that must be mastered by students and taken care of by mathematics teachers in primary school.

In the context of mathematics learning in Malaysia, students are required to solve sentence-based mathematics problems by applying mathematical concepts learned at the end of each topic. Two types of sentence-based mathematics problems are presented when teaching mathematics: routine and non-routine (Wong and Matore, 2020). According to Nurkaeti (2018), routine sentence-based math problems are questions that require the students to solve problems using algorithmic calculations to obtain answers. For non-routine sentence-based math problems, thinking skills and the ability to apply more than one method or solution step are needed by the student to solve the problem (Shawan et al., 2021). According to Rohmah and Sutiarso (2018), problem-solving skills when solving a non-routine sentence-based mathematical problem is a high-level intellectual skill where the students need to use logical thinking and reasoning. This statement also aligns with Wilson's (1997) opinion that solving non-routine sentence-based mathematics always involves high-order thinking skills (HOTS). To solve non-routine and HOTS fundamental sentence-based math problems, a student is required to know various problem-solving strategies for solving the problems (Wong and Matore, 2020). This situation has indirectly made the mastery of sentence-based mathematics problem-solving skills among students more challenging (Mahmud, 2019).

According to Alkhawaldeh and Khasawneh’s findings (2021), the failure of students stems from the teachers’ inability to perform their role effectively in the classroom. This statement is also supported by Abdullah (2020). He argues that the failure of students in mastering non-routine sentence-based mathematics problem-solving skills is due to the teachers rarely supplying these types of questions during the process of learning mathematics in class. A mathematics teacher should consider this issue because the quality of their teaching will affect the students’ mastery level of sentence-based mathematics problem-solving skills.

In addition, the teachers’ efforts to encourage the students to engage in social interactions with the teachers (Jatisunda, 2017) and the teachers’ method of teaching and assessing the level of sentence-based mathematics problem-solving skills (Buschman, 2004) are also challenges that the teachers must face. Strategies that are not appropriate for the students will affect the quality of delivery of the sentence-based mathematics problem-solving skills as well as cause one-way interactions to exist in the classroom. According to Rusdin and Ali (2019), a practical teaching approach plays a vital role in developing the students’ skills when mastering specific knowledge. However, based on previous studies, the main challenges that mathematics teachers face when teaching sentence-based mathematics problem solving are due to the students. These challenges include the students having difficulty understanding sentence-based math problems, lacking knowledge about basic mathematical concepts, not calculating accurately, and not transforming the sentence-based mathematics problems into an operational form (Yoong and Nasri, 2021). This also means that they cannot transform the sentence-based math problems into an operational form (Yoong and Nasri, 2021). As a result, the teacher should diversify his or her teaching strategy by emphasizing understanding the mathematical concepts rather than procedural teaching to reinforce basic mathematical concepts, to encourage the students to work on any practice problems assigned by the teacher before completing any assignments to help them do the calculation correctly, and engaging in the use of effective oral questioning to stimulate student thinking related to the operational need when problem solving. All of these strategies actually help the teachers facilitate and lessen the students’ difficulty understanding sentence-based math problems (Subramaniam et al., 2022).

Meanwhile, Dirgantoro et al. (2019) stated several challenges that the students posed while solving the sentence-based problem. For example, students do not read the questions carefully, the students lack mastery of mathematical concepts, the students solve problems in a hurry due to poor time management, the students are not used to making hypotheses and conclusions, as well as the students, being less skilled at using a scientific calculator. These factors have caused the students to have difficulty mastering sentence-based mathematics problem-solving skills, which goes on to become an inevitable challenge in maths classes. Therefore, teachers need to study these challenges to self-reflect so then their self-professionalism can be further developed (Dirgantoro et al., 2019).

As for the school factor, challenges such as limited teaching resources, a lack of infrastructure facilities, and a large number of students in a class (Rusdin and Ali, 2019) have meant that a conducive learning environment for learning sentence-based mathematics problem-solving skills cannot be created. According to Ersoy (2016), problem-solving skills can be learned if an appropriate learning environment is provided for the students to help them undergo a continuous and systematic problem-solving process.

To develop sentence-based mathematics problem-solving skills among students, various models, pedagogies, activities, etc. have been introduced to assist mathematics teachers in delivering sentence-based mathematics problem-solving skills more effectively (Gurat, 2018; Khoiriyah and Husamah., 2018; Özreçberoğlu and Çağanağa, 2018; Hasibuan et al., 2019). However, students nowadays still face difficulties when trying to master sentence-based mathematics problem-solving skills. This situation occurs due to the lack of studies examining the challenges faced by these mathematics teachers and how teachers use teaching approaches to overcome said challenges. This has led to various issues during the teaching and facilitation of sentence-based mathematics problem-solving skills in mathematics classes. According to Rusdin and Ali (2019), these issues need to be addressed by a teacher wisely so then the quality of teaching can reach the best level. Therefore, mathematics teachers must understand and address these challenges to improve their teaching.

However, so far, not much is known about how primary school mathematics teachers face the challenges encountered when teaching sentence-based mathematics problem-solving skills and what approaches are used to address the challenges in the context of education in Malaysia. Therefore, this study needs to be carried out to help understand the teaching of sentence-based mathematics problem-solving skills in primary schools (Pazin et al., 2022). Due to the challenges when teaching mathematics as stipulated in the Mathematics Curriculum and Assessment Standard Document (Ministry of Education Malaysia, 2020a) which emphasizes mathematical problem-solving skills as one of the main skills that students need to master in comprehensive mathematics learning, this study focuses on identifying the challenges faced by mathematics teachers when teaching sentence-based mathematics problem-solving skills and the approaches that mathematics teachers have used to overcome those challenges. The results of this study can provide information to mathematics teachers to help them understand the challenges when teaching sentence-based mathematics problem-solving skills and the approaches that can be applied to overcome the challenges faced. Therefore, it is very important for this study to be carried out so then all the visions set within the framework of the Malaysian National Mathematics Curriculum can be successfully achieved.

## 2. Conceptual framework

**Figure 1:**
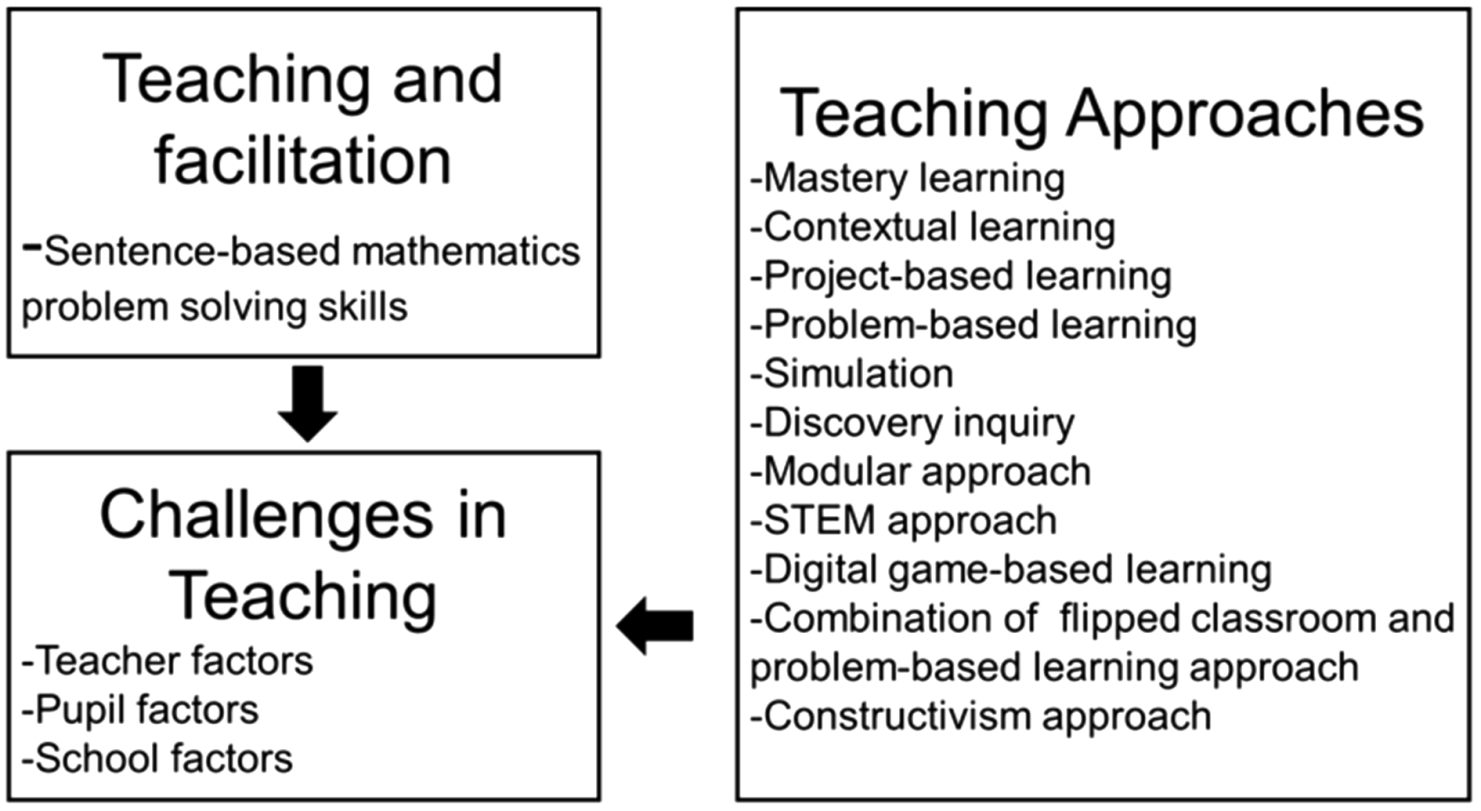
Conceptual framework of the study.

The issue of students lacking mastery of sentence-based mathematics problem-solving skills is closely related to the challenges that teachers face and the teaching approach used. Based on the overall findings of the previous studies, the factors that pose a challenge to teachers when delivering sentence-based mathematics problem-solving skills include challenges from the teacher (Buschman, 2004; Jatisunda, 2017; Abdullah, 2020), challenges from the pupils (Dirgantoro et al., 2019), and challenges from the school (Rusdin and Ali, 2019). As for the teaching approach, previous studies have suggested teaching approaches such as mastery learning, contextual learning, project-based learning, problem-based learning, simulation, discovery inquiry, the modular approach, the STEM approach (Curriculum Development Division, 2019), game-based teaching which uses digital games (Muhamad et al., 2018), and where a combination of the modular approach especially the flipped classroom is applied alongside the problem-based learning approach when teaching sentence-based mathematics problem solving (Alias et al., 2020). This is as well as the constructivism approach (Jatisunda, 2017). The conceptual framework in Figure 1 illustrates that the teachers will face various challenges during the ongoing teaching and facilitation of sentence-based mathematics problem-solving skills.

## 3. Methodology

The objective of this study was to determine the challenges that teachers face while teaching sentence-based mathematics problem-solving skills and the approaches used when teaching those skills. Therefore, a qualitative research approach in the form of a case study was used to collect data from the participants in a Chinese national type of school (SJKC) in Bangsar and Pudu, in the Federal Territory of Kuala Lumpur. The school, SJKC, in the districts of Bangsar and Pudu, was chosen as the location of this study because the school is implementing the School Transformation Program 2025 (TS25). One of the main objectives of the TS25 program is to apply the best teaching concepts and practices so then the quality of the learning and teaching in the classes is improved. Thus, schools that go through the program are believed to be able to diversify their teachers’ teaching and supply more of the data needed to answer the questions of this study. This is because case studies can develop an in-depth description and analysis of the case to be studied (Creswell and Poth, 2018). All data collected through the observations, interviews, audio-visual materials, documents, and reports can be reported on in terms of both depth and detail based on the theme of the case. Therefore, this study collected data related to the challenges and approaches of SJKC mathematics teachers through observations, interviews, and document analysis.

Two primary school mathematics teachers who teach year four mathematics were selected to be the participants of this research using the purposive sampling technique to identify the challenges faced and the approaches used to overcome those challenges. The number of research participants in this study was sufficient enough to allow the researcher to explore the real picture of the challenges found when teaching sentence-based mathematics problem-solving skills and the approaches that can be applied when teaching to overcome the challenges faced. According to Creswell and Creswell (2018), the small number of study participants is sufficient when considering that the main purpose of the study is to obtain findings that can give a holistic and meaningful picture of the teaching and learning process in the classroom. However, based on the data analysis for both study participants, the researcher considered repeated information until it reached a saturation point. The characteristics of the study participants required when they were supplying the information for this study were as follows:

- New or experienced teachers.
- Year four math teacher.
- Teachers teach in primary schools.
- The teacher teaches the topic of sentence-based mathematics problem-solving skills.

The types of instruments used in the study were the observation protocol, field notes, interview protocol, and participants’ documents. In this study, the researcher used participatory type observations to observe the teaching style of the teachers when engaged in sentence-based mathematics problem-solving skills lessons. Before conducting the study, the researcher obtained consent to conduct the study from the school as well as informed consent from the study participants to observe their teaching. During the observation, the teacher’s teaching process was recorded and transcribed using the field notes provided. Then, the study participants submitted and validated the field notes to avoid biased data. After that, the field notes were analyzed based on the observation protocol to identify the teachers’ challenges and teaching approaches in relation to sentence-based mathematics problem-solving skills. Throughout the observation process of this study, the researcher observed the teaching of mathematics teachers online at least four times during the 2 months of the data collection at the research location.

Semi-structured interviews were used to identify the teachers’ perspectives and views on teaching sentence-based mathematics problem-solving skills in terms of the challenges faced when teaching sentence-based mathematics problem-solving skills and the approaches used by the teachers to overcome those challenges. To ensure that the interview data collected could answer the research questions, an interview protocol was prepared so then the required data could be collected from the study participants (Cohen et al., 2007). Two experts validated the interview protocol, and a pilot study was conducted to ensure that the questions were easy to understand and would obtain the necessary data. Before the interview sessions began, the participants were informed of their rights and of the related research ethics. Throughout the interview sessions, the participants were asked two questions, namely:

1. What are the challenges faced when teaching mathematical problem-solving skills earlier?
2. What teaching approaches are used by teachers when facing these challenges? Why?

Semi-structured interviews were used to interview the study participants for 30 min every interview session. The timing ensured sufficient time for both parties to complete the question-and-answer process. Finally, the entire interview process was recorded in audio form. The audio recordings were then transcribed into text form and verified by the study participants.

The types of document collected in this study included informal documents, namely the daily lesson plan documents of the study participants, the work of the students of the study participants, and any teaching aids used. All of the documents were analyzed and used to ensure that the triangulation of the data occurred between the data collected from observations, interviews, and document analysis.

**Figure 2:**
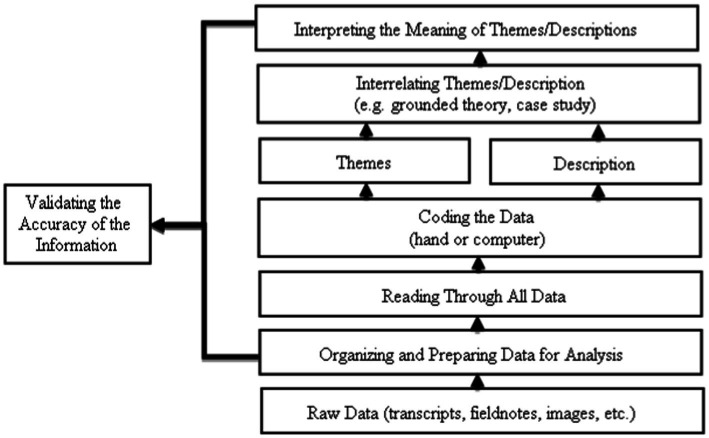
Data analysis steps (Creswell and Creswell, 2018).

All data collected through the observations, interviews, and documentary analyses were entered into the NVIVO 11 software to ensure that the coding process took place simultaneously. The data in this study were analyzed using the constant comparative analysis method including open coding, axial coding, and selective coding to obtain the themes and subthemes related to the focus of the study (Kolb, 2012). The NVIVO 11 software was also used to manage the data stack obtained from the interviews, observations, and document analysis during the data analysis process itself. In order to ensure that the themes generated from all of the data were accurate, the researcher carried out a repetitive reading process. The process of theme development involved numerous steps. First, the researcher examined the verbatim instruction data several times while looking for statements or paragraphs that could summarize a theme in a nutshell. This process had already been completed during the verbatim formation process of the teaching, while preparing the transcription. Second, the researcher kept reading (either from the same or different data), and if the researcher found a sentence that painted a similar picture to the theme that had been developed, the sentence was added to the same theme. This process is called “pattern matching” because the coding of the sentences refers to the existing categories (Yin, 2003). Third, if the identified sentence was incompatible with an existing theme, a new theme was created. Fourth, this coding procedure continued throughout each data set’s theme analysis. The repeated reading process was used to select sentences able to explain the theme or help establish a new one. In short, the researcher conducted the data analysis process based on the data analysis steps proposed by Creswell and Creswell (2018), as shown in Figure 2.

## 4. Findings

**Figure 3:**
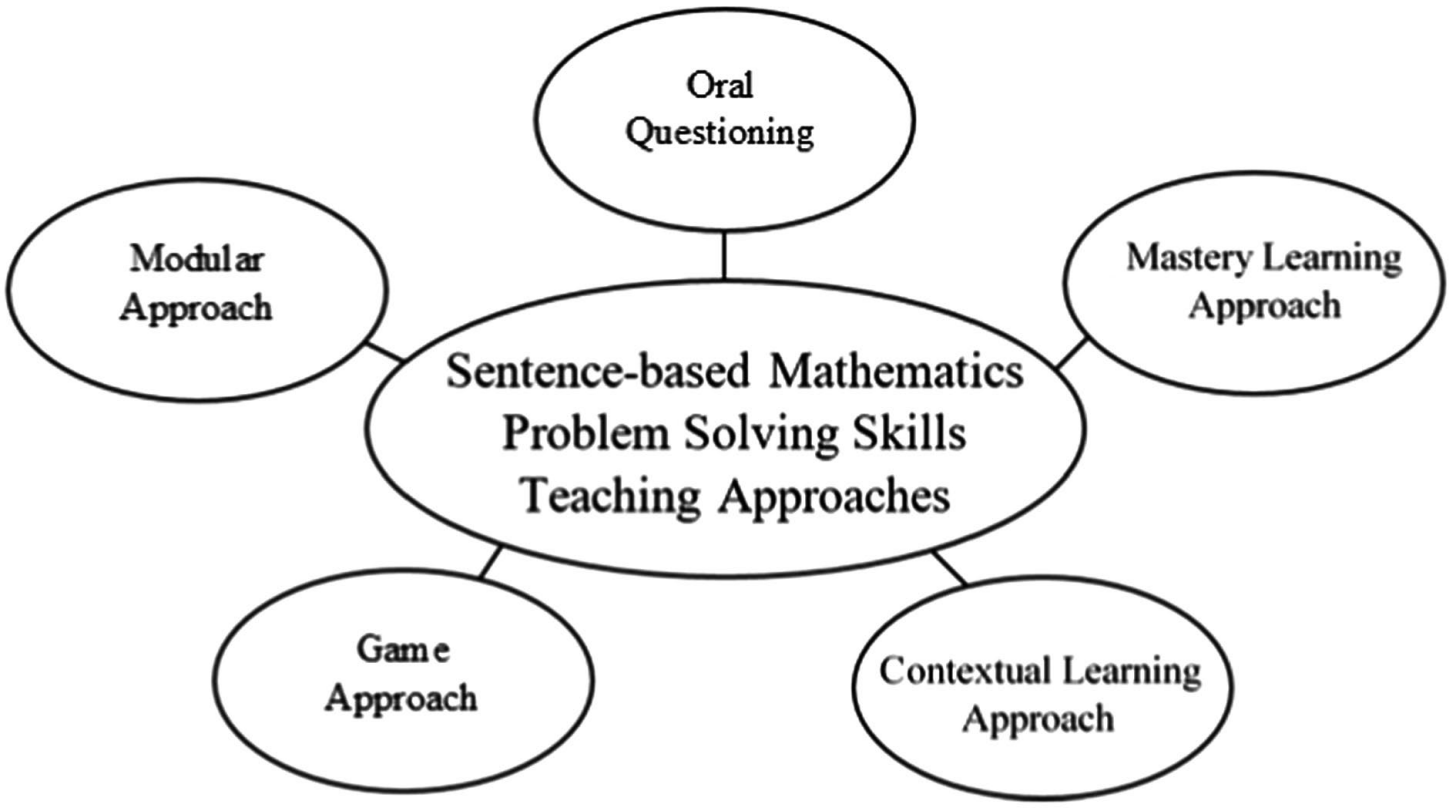
Sentence-based mathematics problem-solving teaching approaches.

The findings of this study are presented based on the objective of the study, which was to identify the challenges faced by teachers and the approaches used to addressing those challenges when imparting sentence-based mathematics problem-solving skills to students in year four. Several themes were formed based on the analysis of the field notes, interview transcripts, and daily lesson plans of the study participants. This study found that teachers will face challenges that stem from the readiness of students to master sentence-based mathematics problem-solving skills, the teachers’ teaching style, and the equipment used for delivering sentence-based mathematics problem-solving skills. Due to facing these challenges, teachers have diversified their teaching approaches (Figure 3; Table 1).

**Table 1:** Teacher challenges when teaching sentence-based mathematics problem-solving skills.

**Theme**	**Subtheme**	**Evidence**			
Low mastery skills in the students	Low mastery of reading skills	**Teacher interview transcript**			
		- Pupils cannot read and understand mathematical questions in the form of text because the student’s language foundation is not good. (TB1/GM 1: 12)			
		**Field notes**			
		- Pupil K read intermittently, and the teacher gave guidance. (NL2/GM 1: 38–39)			
		- Pupil Z pauses in the process of reading the mathematics question. (NL1/GM 2: 5–7)			
		**Student interview transcript**			
		I have a lot of unrecognizable words, and my Chinese is bad. English is easier than Chinese. (TB/MK: 38)			
	Low mastery of the medium of instruction	**Teacher interview transcript**			
		- Pupils are not able to understand the question or interpret the question in depth. (TB 1/GM 2: 10)			
		**Field notes**			
		- Pupil C has raised his problem that he still does not understand this question. (NL1/GM 1: 33–34)			
		- The teacher conducts the second explanation of this question to guide students to understand the question through a question-and-answer session. (NL1/GM 1: 35–39)			
		**Student interview transcript**			
		I do not understand what this question means. (TB/MZ: 18)			
	Low mastery of mathematical concepts	**Teacher interview transcript**			
		- Lack of mathematical knowledge also encourages students to make mistakes in choosing strategies, especially to solve sentence-length mathematical problems, and is unclear in choosing the appropriate strategy to apply. (TB1/GM 2: 12)			
		**Field notes**			
		- Pupil W continues to object to pupil L’s statement and explains that not the teacher does not allow it. The teacher has explained that the writing is incorrect because the question is multiplied by three instead of 3 days. (NL3/GM 1: 30–33)			
		**Student work**			
		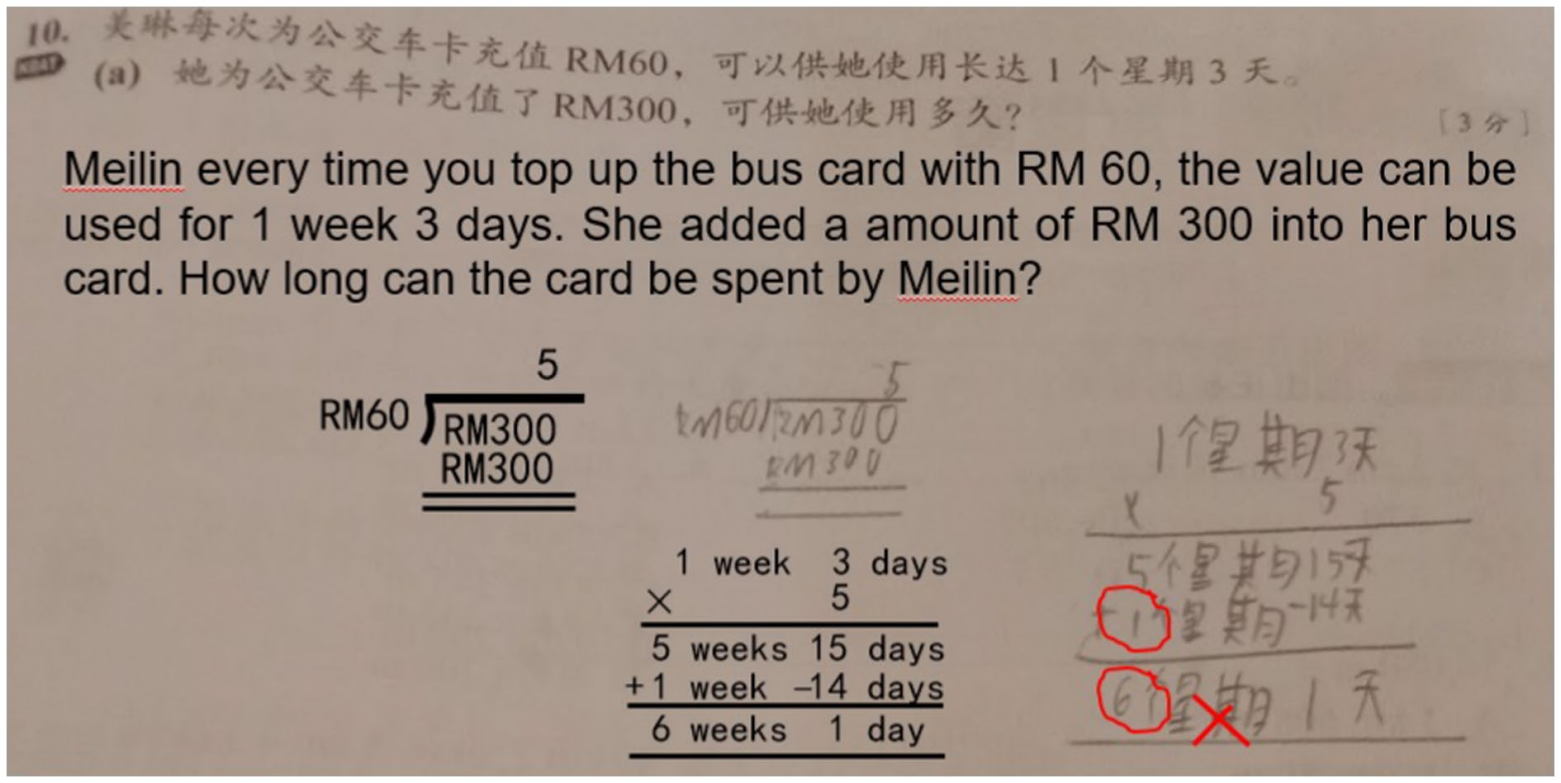			
		Error in the concept of unit conversion where 1 week has 14 days			
Insufficient teaching time	Teaching approach used	**Teacher interview transcript**			
		- But the repeating method in teaching a topic will delay my teaching progress. (TB 1/GM 1: 24)			
		**Daily lesson plan analysis**			
		Participants’ daily lesson plan analysis			
		**Participants**	**Topic**	**Period in teaching problem solving**	**Weeks**
		GM 1	4 – Time	06.09.2021–04.10.2021 (Sem Break 11.09.2021–19.09.2021)	3
		GM 2	5 – Size and Measurements	27.09.2021–08.10.2021	2
		**Annual mathematics lesson plan analysis**			
		Annual mathematics lesson plan			
		13 20.09.21/24.09.21	Title: 5.0 SIZE AND MEASUREMENT Standard content: 5.3 Volume of liquid	Students can: 5.3.1 Solve mathematical sentences to determine the combined operations of adding and subtracting volumes of liquids involving units in milliliters and liters 5.3.2 Solve mathematical sentences through the combined operations of multiplying and dividing the volume of a liquid involving units in milliliters and liters.	Note: Introduce the unit of liquid volume to the imperial measurement system: Gelen (gal) Quarter (qt) Pain (pt) Activity suggestion: - Use objects and software for the conversion of units into a volume measurement. - Use a variety of computational strategies to solve mathematical sentences.
		14–15 27.09.21/08.10.21	Title: 5.0 SIZE AND MEASUREMENT Standard content: 5.4 Problem Solving	Students can: 5.4.1 Solve problems related to measurements in everyday situations.	Activity suggestion: Use the troubleshooting steps below: Understand the problem. Plan solution strategies. Implement strategies. Check the answers. - Use various problem-solving strategies such as reasoning logically and identifying patterns. - Use various teaching strategies such as simulations, STEM, and modular approaches.
Lack of infrastructure (ICT)	Unstable internet connection	**Field notes**			
		- Pupil H has responded that the whiteboard provided by the teacher through the link provided does not work. (NL 3/GM 1: 24–25)			
		- Pupil W took a long time, did not respond, and dropped out of class activities. (NL 4/GM 1: 50–51)			

## 5. Discussion

### 5.1. Challenges for teachers when imparting sentence-based mathematics problem-solving skills

A mathematics teacher will face three challenges when teaching sentence-based mathematics problem-solving skills. The first challenge stems from the low mastery skills held by a student. Pupils can fail to solve sentence-based mathematics problems because they have poor reading skills, there is a poor medium of instruction used, or they have a poor mastery of mathematical concepts (Johari et al., 2022). This indicates that students who are not ready or reach a minimum level of proficiency in a language, comprehension, mathematical concepts, and calculations will result in them not being able to solve sentence-based mathematics problems smoothly.

These findings are consistent with the findings of the studies by Raifana et al. (2016) and Dirgantoro et al. (2019) who showed that students who are unprepared in terms of language skills, comprehension, mathematical concepts, and calculations are likely to make mistakes when solving sentence-based mathematics problems. If these challenges are not faced well, the students will become passive and not interact when learning sentence-based mathematics problem-solving skills. This situation occurs because students who frequently make mistakes will incur low self-confidence in mathematics (Jailani et al., 2017). This situation should be avoided by teachers and social interaction should be encouraged during the learning process because the interaction between students and teachers can ensure that the learning outcomes are achieved by the students optimally (Jatisunda, 2017).

The next challenge stems from the teacher-teaching factor. This study found that how teachers convey problem-solving skills has been challenging in terms of ensuring that their students master sentence-based mathematics problem-solving skills (Nang et al., 2022). The mastery teaching approach has caused the teaching time spent on mathematical content to be insufficient. Based on the findings of this study, the allocation of time spent ensuring that the students master the skills of solving sentence-based mathematics problems through a mastery approach has caused the teaching process not to follow the rate set in the annual lesson plan.

In this study, the participants spent a long time correcting the students’ mathematical concepts and allowing students to apply the skills learned. The actions of the participants of this study are in line with the statement of Adam and Halim (2019) that teachers need more time to arouse their students’ curiosity and ensure that students understand the correct ideas and concepts before doing more challenging activities. However, this approach has indirectly posed challenges regarding time allocation and ensuring that the students master the skills of sentence-based mathematics problem-solving. Aside from ensuring that the students’ master problem-solving skills, the participants must also complete the syllabus set in the annual lesson plan.

Finally, teachers also face challenges in terms of the lack of information and communication technology (ICT) infrastructure when implementing the teaching and facilitation of sentence-based mathematics problem-solving skills. In this study, mathematics teachers were found to face challenges caused by an unstable internet connection such as the problem of their students dropping out of class activities and whiteboard links not working. These problems have caused one mathematics class to run poorly (Mahmud and Law, 2022). Throughout the implementation of teaching and its facilitation, ICT infrastructure equipment in terms of hardware, software, and internet services has become an element that will affect the effectiveness of virtual teaching (Saifudin and Hamzah, 2021). In this regard, a mathematics teacher must be wise when selecting a teaching approach and diversifying the learning activities to implement a suitable mathematics class for students such as systematically using tables, charts, or lists, creating digital simulations, using analogies, working back over the work, involving reasoning activities and logic, and using various new applications such as Geogebra and Kahoot to help enable their students’ understanding.

### 5.2. Teaching sentence-based mathematics problem-solving skills—Approaches

In this study, various approaches have been used by the teachers facing challenges while imparting sentence-based mathematics problem-solving skills. Among the approaches that the mathematics teachers have used when teaching problem-solving skills are the oral questioning approach, mastery learning approach, contextual learning approach, game approach, and modular approach. This situation has shown that mathematics teachers have diversified their teaching approaches when facing the challenges associated with teaching sentence-based mathematics problem-solving skills. This action is also in line with the excellent teaching and facilitation of mathematics proposal in the Curriculum and Assessment Standards Document revised KSSR Mathematics Year 4 (Curriculum Development Division, 2019), stating that teaching activities should be carefully planned by the teachers and combine a variety of approaches that allow the students not only to understand the content in depth but also to think at a higher level. Therefore, a teacher needs to ensure that this teaching approach is applied when teaching sentence-based mathematics problem-solving skills so then the students can learn sentence-based mathematics problem-solving teaching skills in a more fun, meaningful, and challenging environment (Mahmud et al., 2022).

Through the findings of this study, the teaching approach used by mathematics teachers was found to have a specific purpose, namely facing the challenges associated with teaching sentence-based mathematics problem-solving skills in the classroom. First of all, the oral questioning approach has been used by teachers facing the challenge of students having a poor understanding of the medium of instruction. The participants stated that questioning the students in stages can guide them to understanding the question and helping them plan appropriate problem-solving strategies. This opinion is also supported by Maat (2015) who stated that low-level oral questions could help the students achieve a minimum level of understanding, in particular remembering, and strengthening abstract mathematical concepts. The teacher’s action of guiding the students when solving sentence-based mathematics problems through oral questioning has ensured that the learning takes place in a student-centered manner, providing opportunities for the students to think and solve problems independently (Mahmud and Yunus, 2018). This action is highly encouraged because teaching mathematics through the conventional approach is only effective for a short period, as the students can lack an understanding or fail to remember the mathematical concepts presented by the teacher (Ali et al., 2021).

In addition, this study also found that the participants used the mastery approach to overcome the challenges of poor reading skills and poor mastery of mathematical concepts among the students. The mastery approach was used because it can provide more opportunities and time for the students to improve their reading skills and mastery of mathematical concepts (Shawan et al., 2021). This approach has ensured that all students achieve the teaching objectives and that the teachers have time to provide enrichment and rehabilitation to the students as part of mastering the basic skills needed to solve sentence-based mathematics problems. This approach is very effective at adapting students to solving sentence-based mathematics problems according to the solution steps of the Polya model as well as the mathematical concepts learned in relation to a particular topic. The finding is in line with Ranggoana et al. (2018) and Mahmud (2019) study, which has shown that teaching through a mastery approach can enhance the student’s learning activities. This situation clearly shows that the mastery approach has ensured that the students have time to learn at their own pace, where they often try to emulate the solution shown by the teacher to solve a sentence-based mathematics problem.

Besides that, this study also found that mathematics teachers apply contextual learning approaches when teaching and facilitating sentence-based mathematics problem-solving skills. In this study, mathematics teachers have linked non-routine problems with examples from everyday life to guide the students with poor language literacy to help them understand non-routine problems and plan appropriate solution strategies. Such relationships can help the students process non-routine problems or mathematical concepts in a more meaningful context where the problem is relevant to real situations (Siew et al., 2016). This situation can develop the students’ skill of solving sentence-based math problems where they can choose the right solution strategy to solve a non-routine problem. This finding is consistent with the results of Afni and Hartono (2020). They showed that the contextual approach applied in learning could guide the students in determining appropriate strategies for solving sentence-based math problems. These findings are also supported by Seliaman and Dollah (2018) who stated that the practice of teachers giving examples that exist around the students and in real situations could make teaching and the subject facilitation easier to understand and fun.

Furthermore, the game approach was also used by the participants when imparting sentence-based mathematics problem-solving skills. According to Sari et al. (2018), the game approach to teaching mathematics can improve the student learning outcomes because the game approach facilitates the learning process and provides a more enjoyable learning environment for achieving the learning objectives. In this study, the game approach was used by the teachers to overcome the challenge of mastering the concept of unit conversion, which was not strong among the students (Tobias et al., 2015; Hui and Mahmud, 2022). The participants used the game approach to teach induction sets that guided the students in recalling mathematical concepts. The action provided a fun learning environment and attracted the students to learning mathematical concepts, especially in the beginning of the class. This situation is consistent with the findings of Muhamad et al. (2018). They showed that the game approach improved the students’ problem-solving skills, interests, and motivation to find a solution to the problem.

Regarding the challenge of insufficient teaching time and a lack of ICT infrastructure, modular approaches such as flipped classrooms have been used to encourage students to learn in a situation that focuses on self-development (UNESCO, 2020). In this study, the participants used instructional videos with related content, clear instructions, and worksheets as part of the Google classroom learning platform. The students can follow the instructions to engage in revision or self-paced learning in their spare time. This modular approach has ensured that teachers can deliver mathematical content and increase the effectiveness of learning a skill (Alias et al., 2020). For students with unstable internet connections, the participants have used a modular approach to ensure that the students continue learning and send work through other channels such as WhatsApp, by email, or as a hand-in hardcopy. In short, an appropriate teaching approach needs to be planned and implemented by the mathematics teachers to help students master sentence-based mathematics problem-solving skills.

## 6. Conclusion

Overall, this study has expanded the literature related to the challenges when teaching sentence-based mathematics problem-solving skills and the approaches that can be applied while teaching to overcome the challenges faced. This study has shown that students have difficulty mastering sentence-based mathematics problem-solving skills because they do not achieve the minimum mastery of factual knowledge, procedural skills, conceptual understanding, and the ability to choose appropriate strategies (Collins and Stevens, 1983). This situation needs to be taken into account because sentence-based mathematics problem-solving skills train the students to always be prepared to deal with problems that they will be faced with in their daily life. Through this study, teachers were found to play an essential role in overcoming the challenges faced by choosing the most appropriate teaching approach (Baul and Mahmud, 2021). An appropriate teaching approach can improve the students’ sentence-based mathematics problem-solving skills (Wulandari et al., 2020). Teachers need to work hard to equip themselves with varied knowledge and skills to ensure that sentence-based mathematics problem-solving skills can be delivered to the students more effectively. Finally, the findings of this study were part of obtaining extensive data regarding the challenges that mathematics teachers face when teaching sentence-based mathematics problem-solving skills and the approaches used to address those challenges in the process of teaching mathematics. It is suggested that a quantitative study be conducted to find out whether the findings obtained can be generalized to other populations. This is because this study is a qualitative one, and the findings of this study cannot be generalized to other populations.

The findings of this study can be used as a reference to develop the professionalism of mathematics teachers when teaching mathematical problem-solving skills. However, the study’s findings, due to being formulated from a small sample size, cannot be generalized to all mathematics teachers in Malaysia. Further studies are proposed to involve more respondents to better understand the different challenges and approaches used when teaching sentence-based mathematics problem-solving skills.

## Data availability statement

The raw data supporting the conclusions of this article will be made available by the authors, without undue reservation.

## Ethics statement

This study was reviewed and approved by The Malaysian Ministry of Education. The participants provided their written informed consent to participate in this study.

## Author contributions

AL conceived and designed the study, collected and organized the database, and performed the analysis. AL and MM co-wrote the manuscript and contributed to manuscript revision. All authors read and approved the final submitted version.

## Funding

The publication of this article is fully sponsored by the Faculty of Education Universiti Kebangsaan Malaysia and University Research Grant: GUP-2022-030, GGPM-2021-014, and GG-2022-022.

## Conflict of interest

The authors declare that the research was conducted in the absence of any commercial or financial relationships that could be construed as a potential conflict of interest.

## Publisher’s note

All claims expressed in this article are solely those of the authors and do not necessarily represent those of their affiliated organizations, or those of the publisher, the editors and the reviewers. Any product that may be evaluated in this article, or claim that may be made by its manufacturer, is not guaranteed or endorsed by the publisher.
